# Improving community based AEFI (Adverse Events Following Immunization) reporting rate through telephone “beep” in a Cameroon health district: a randomized field trial

**DOI:** 10.11604/pamj.2015.22.351.8368

**Published:** 2015-12-11

**Authors:** Marcellin Tsafack, Jérôme Ateudjieu

**Affiliations:** 1 Department of Biomedical Sciences, University of Dschang, Dschang, Cameroon; 2 Division of Health Operations Research, Ministry of Public Health, Cameroon; 3 MA Santé (Meilleur Accès aux soins de santé), Yaoundé, Cameroun

**Keywords:** EPI, telephone “beep”, AEFI, Cameroon

## Abstract

**Introduction:**

AEFIs underreporting is one of different barriers to achieving objectives of pharmaco vigilance of vaccine worldwide. Studies describe it as being related to limited awareness of health personnel and of vaccinees or of their parents. The objective was to assess the effect of telephone “beep” on community based reporting rates of AEFIs during routine immunization sessions in a Cameroon Health District.

**Methods:**

It was a randomized control trial implemented during routine EPI in Biyem-Assi health district (Cameroon). Parents of vaccinated children were randomly assigned: i) to receive the telephone contact of the investigation team and was advised to ‘'beep’‘(short phone call not picked up) the investigators team in the case any medical incidence occurs within the 30 days following the immunization (intervention group) or; ii) to return to the health facility in case any medical incidence occurs within the same period (control group). The main outcome was AEFI incidence rate.

**Results:**

236 parents were assigned to the intervention group and 235 to the control group. Of 1192 doses of EPI vaccines administered, 20 AEFIs (392 AEFIs/100000 doses/week) were reported within 30 days after vaccine administration. These included 19 (829 AEFIs/100000 doses/week) AEFIs in the intervention group and 1 (43 AEFIs/100000 doses/week) AEFI in the control group. The AEFIs reporting rate in the intervention group was significantly higher than that in the control group [RR = 18.9; CI95 (2.5; 140.0) (P=0.0004)].

**Conclusion:**

The use of telephone “beep” significantly increases at affordable cost community based AEFI reporting rate in routine EPI.

## Introduction

Adverse events following immunization (AEFI) surveillance is a recommendation for the Expanded Program on Immunization (EPI) at national and international levels [1–5]. However it is limited due to low reporting rate, often explained by many reasons [6–8]. The situation is more serious in the developing countries due to limited resources, geographical and cultural inaccessibility to health facilities [9–11]. Health facility based AEFI detection and reporting by health personnel is currently recommended in the Cameroon national EPI SOPs [12]. However, AEFI reporting forms are rarely filled and transmitted through the right channel as required. This situation has not yet been investigated but may can be explained by weakness of the surveillance system and inaccessibility of health facilities to populations due to lack of awareness, enclave and believes of the population [11–16]. Several strategies have been tested to improve the AEFI detection and reporting rates including standardized health facility supervision [17] and SMS reminder [17–20]. In a study in Cameroon, 451 health facilities were randomly assigned to receive either weekly standardized “short message service” (sms) text messages or a weekly standardized supervisory visits or no intervention for four weeks after immunization campaign. Furthermore, a study in Australia for five months a cohort of 3,047 pregnant women who received the 2013 trivalent influenza vaccine (TIV)) provided mobile telephone numbers and were sent sms inquiring whether they had experienced an AEFI [18]. Moreover, in another study in the united states (US), for 19 months 3226 adults and parents of paediatric patients who received routine vaccination were sent an sms by smart vax, a prototypic active monitoring system for AEFI, inquiring whether they had experience an AEFI [19]. Though beneficial to an extent, their effectiveness is limited since certain strategies had no significant effect or no control strategy. Besides, all these strategies are very costly strategy is needed to ensure universal benefit. We believed that using telephone “beep” is less costly and would increase reporting rate of AEFI, higher than the reporting rate of the routine AEFI surveillance activities. This study was conducted to assess during routine immunization in a health district in Cameroon, the effect of telephone “beep” on community based AEFI reporting rate.

## Methods

### Ethical statement

An ethical approval was obtained from the national ethics committee of Cameroon on health research before implementation of the study (N° 2013/11/389/L/CNERSH/SP)

### Study design

This was a randomized controlled field trial. Parents meeting inclusion criteria were randomly assigned to receive (i) a telephone contact of the investigation team and was advised to call back in the case any medical incidence occurs within the 30 days following the immunization or (ii) was advised as routinely recommended to return to the health facility in case any medical event occurs during the indicated period. The primary outcome was the incidence of AEFI per 100 parents per month reported. Informed consent of all participants was obtained after the procedure and objective of the study explained to them.

### Target Population

The study targeted parents coming with their children for vaccination to the district hospital of Biyem-Assi-Yaoundé, these parents were eligible for the study. Inclusion criteria for parents in the study included possession of a mobile phone and accepting to participate.

### Interventions

#### Intervention

Mobile phone signal or “beep”: Parents in the interventional group received a telephone contact of the investigation team and advised to call back in the case any medical event occurs within 30 days following the immunization

#### Control

Parents in the control group receive the routine advice as required to come back in the health facility in case any event occurs during the indicated period after immunization

#### Outcome

The primary outcome was the incidence of AEFI per 100 parents per month reported during intervention period. The numerator being the sum of detected and reported AEFI and the denominator the number of parents multiplied by the number of month of follow-up. Information concerning the telephone number of parents, the age, sex and type of vaccine taken by the child were collected in a follow-up grid.

### Sample size

Sample size calculation was based on the test of null hypothesis that there is no difference between telephone “beep” usage on AEFI reporting rate compared to returning to the health facility as routinely advised. The level of significance was set at 0.05. The sample size was calculated using the Oxford university press publications on methods for field trial interventions against tropical diseases. Assuming telephone “beep” increases reporting to 50%,with the relative reporting rate ratio (RR) as 1.5. To account for 20% drop out rate, the sample size was increased to 447.

### Randomization

A restrictive randomization was used. It was an allocation with a block size of two and two interventions named A and B. Therefore, four allocation sequences were possible (AA, AB, BA, BB). Each allocation sequence was numbered from one to four and using a table of random numbers, for each 2 participants, a number was randomly selected to choose the appropriate sequence. The random allocated sequence was generated by the principal investigator; the enrollment and assignation of participants were done by the investigation team (principal investigator and two master students in public health). Blinding here was not necessary because the study obliged to the participants or investigator to know group belongings.

### Statistical analysis

The incidence rate of reported AEFI was estimated per study group and measured by estimating the number of AEFI detected and reported per 100parents per month. The effect or the difference in reporting rates in the both groups were estimated by calculating the relative incidence rate (RR). The chi-squared test permitted us to compare reporting rates and the p value indicated the significance of the effect (degree of significance fixed at 5%). All analysis was done by the software epi-infos 3.5.3 version.

## Results

### Recruitment and participants flow

**Figure 1:**
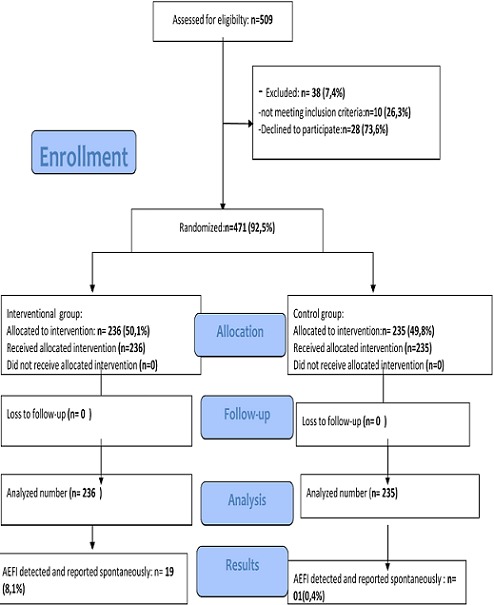
Participants flow diagram at district hospital of Biyem-Assi (Cameroon), from December 2013 to February 2014

From the 23/12/2013 up to the 23/01/2014 a total of the 509 parents were asked to participate to the study 38(7.4%) were excluded; reason for exclusion was essentially absence of cell phone. A total of 471 (92.5%) parents were included and assigned to each of the two groups, 236(50.1%) and 235 (49.8%) in the interventional and control group respectively. The same proportions received intended interventions and were analyzed for AEFI reporting. Figure 1 shows the enrollment and assignment of participants in the flow. The vaccine against poliomyelitis, the pentavalent (DPT-HepB+Hib) and the pneumo-13 were vaccines mostly administered. Apart from the intervention there was no statistical significant difference between the two groups concerning the sex and the age of children. Table 1 shows the sex, age and vaccine administered to participants by study groups. 1192 doses of vaccines were administered to 471 children.

**Table 1:** Sex, age and vaccine administered to participants by study groups

Caracteristic		Beep Group		Non- beep Group		P value
	E	E (n)	P (%)	E (n)	P (%)	
Sex	Male	118	50.0	126	53.6	0.43
	Female	118	50.0	109	46.4	0.43
Age group(in weeks)	< 1	40	16.9	43	18.3	0.70
	[2-6]	55	23.3	45	19.1	0.20
	[7-10]	45	19.1	42	17.9	0.64
	[11-14]	37	15.7	51	21.1	0.09
	[15-52]	45	19.1	48	20.4	0.71
	> 52	14	5.9	06	2.6	0.06
Vaccine	BCG	40	16.9	46	19.6	0.60
	OPV	177	75	180	76.9	0.62
	PENTA	137	58.1	135	57.4	0.89
	Pneumo	136	57.6	135	57.4	0.96
	MV	45	19.1	48	20.4	0.71
	YFV	45	19.1	48	20.4	0.71

BCG: Bacille de Calmette et Guérin - E: Effectif - P: percentage - OPV: 386 Oral Poliomyelitis Vaccine- PENTA: Diphteria, pertusis, Tetanus, Hepatitis B, Haemophilus- YFV: Yellow fever vaccine - MV: Measles Vaccine

### Outcomes and risk estimations

For one month of follow-up, a total of 20(4.2%) AEFI have been detected and reported spontaneously, 19 in interventional group and 01in the control group. The reporting rate in the interventional group (8.1%/month) was significantly higher than the rate in the control group (0.4%/month), [RR = 18.9; CI 95 (2.5; 140.0) (P = 0.0004)]. The attributable risk was 7.6% and the attributable fraction 0.94. Nine (45%) of AEFI were local reactions at the site of injection and 7(35%) fever.

## Discussion

Our study suffers from bias and limits. The number of participants included in the survey was limited. The effect of telephone “beep” on the reporting rate of AEFI was gotten after a study on a small sample. It would be difficult to extrapolate this result to the region or the country. Our study suffers from bias and limits. The number of participants included in the survey was limited. Furthermore all participants were recruited in the district hospital of Biyem-Assi-Yaoundé. Is possible that the majority of participants were residents of an urban zone and therefore more accustomed to the use of the telephone “beep”. It could have introduced a selection bias because it would be difficult to say with certainty that rural zone residents will respond likely to those of the urban zones concerning the use of telephone “beep” for the surveillance of the AEFI. The reporting rate in the interventional group was significantly higher than the rate in the control group, (P = 0.0004)]. The interest of this study is to identify an intervention as the use of the telephone “beep” to improve the community based AEFI reporting rate. In Cameroon, mobile phone operators cover the 10 regions of the country. “Mobile Telephone Network” (MTN) covers 84% of the population and orange 90% [21].

To the best of our knowledge, no study has been done on the reporting of AEFI based on the use of telephone “beep”. Nevertheless, other strategies tested us were revealed beneficial. A study using SMS for AEFI reporting was done in Cambodia. For 132 SMS sent, AEFI reporting rate for 51 days was 17.4% [20]. This rate is superior to the one we got in our study concerning interventional group (8.4%). The difference in composition and in number of our samples could explain this difference. Another study testing automated text messages to monitor AEFI, produced results that are in line with the one obtained by this study. The proportion of respondent who reported possible AEFI was 11.3% [19]. But comparably to our study, there was no significant difference in proportion of reported AEFI between patients who replied by sms and those who did not respond by sms but were subsequently contacted by a telephone. Mobile phone based tools like sms have the capacity to complement existing passive reporting systems but the implication of a cost could be a barrier to reporting compared to our strategy. The absence of a control group during sms studies also show limits in proving the contribution of sms to improve rate of AEFI reporting, which was taken into account in our study. Another strategy was tested in Cameroon, concerning the effect of supervision on reporting rate. Supervision was more effective than sms or routine surveillance activities in improving AEFI reporting rate [17]. This strategy though beneficial, there is weakness of the health system in our context to conduct supervision. The use of telephone “bip” or signal easy to learn and to use or implement compared to supervision is an added value. Considering the limits of this study, it reveals that the use of telephone “bip” increases significantly community based AEFI reporting rate and can be used in Cameroon where the system of pharmaco vigilance needs an improvement. This strategy, free of charge, easy to use and to learn, simple compared to other strategies, could render this one best for community based AEFI reporting rate.

## Conclusion

The use of telephone “bip” increases significantly community based AEFI reporting rate,[RR = 18.9; CI95 (2.5; 140.0) (p=0.0004)]. This strategy could be tested to a larger scale and recommended as a surveillance tool of AEFI and adverse effects of drugs on Cameroonian market and why not apply for surveillance of community based diseases.
